# Walshe’s Physicians’ Combined Call Book and Tablet

**Published:** 1881-11-20

**Authors:** 


WALSHE'S Physicians' Combined Call Book and Tablet—sixth edi tion—very neat, and in all respects convenient, containing the various tables, abbreviations, doses, formulæ—antidotes to poisons, disinfectants, obstetric tables, incompatibles, calendar, call lists, general memoranda, etc., etc. Published by Ralph Walshe, 332 C. St., Washington, D. C. Price, $1.50.

